# 2-Stage and 3-stage restorative proctocolectomy with ileal pouch-anal anastomosis for ulcerative colitis show comparable short- and long-term outcomes

**DOI:** 10.1038/s41598-024-72466-0

**Published:** 2024-10-01

**Authors:** Maximilian Brunner, Martin Brandl, Axel Denz, Christian Krautz, Georg F. Weber, Robert Grützmann, Klaus Weber

**Affiliations:** Department of General and Visceral Surgery, Friedrich-Alexander-University, Krankenhausstraße 12, 91054 Erlangen, Germany

**Keywords:** Ulcerative colitis, Proctocolectomy, Ileal pouch-anal anastomosis, Outcome, Pouch failure, Gastrointestinal diseases, Outcomes research

## Abstract

Restorative proctocolectomy with ileal pouch-anal anastomosis (IPAA) represents the standard treatment for therapy-refractory, malignant or complicated ulcerative colitis (UC) and can be performed as a 2-stage or 3-stage procedure. This study aimed to compare the short- and long-term outcomes after 2- and 3-stage IPAA in patients with UC in our department. A retrospective analysis of 176 patients with UC who received 2- or 3-stage restorative proctocolectomy with IPAA at our institution from 2001 to 2021 was performed. Outcomes for short-term (morbidity, longer hospital stay, readmission) and long-term (pouch failure and quality of life) parameters were compared between the 2- and 3-stage procedure. Regarding short-term outcomes for all patients, in-hospital morbidity and readmission rates after any surgical stage were observed in 69% and 24%, respectively. Morbidity and readmission did not differ significantly between the 2- and 3-stage procedure in uni- and multivariate analysis. Median length of hospital stay for all stages was 17 days. The 3-stage procedure was identified as an independent factor for longer hospital stay (OR 3.8 (CI 1.3–10.8), *p* = 0.014). Pouch failure and failure of improved quality of life during long-term follow-up occurred both in 10% of patients, with no significant differences between the 2- and 3-stage procedure in uni- and multivariate analysis. Our data suggest that both the 2- and 3-stage proctocolectomy with IPAA demonstrate favourable and comparable postoperative short- and long-term outcomes, with a high rate of improved quality of life in patients with UC.

## Introduction

Despite constant improvements in conservative therapy—in particular due to the widespread use and continuous development of biologicals—about 10–15% of all patients with ulcerative colitis require a surgical procedure, most commonly due to refractory disease to conservative therapy measures or due to dysplasia or malignancy^1–4^. The standard surgical procedure is proctocolectomy with ileal pouch-anal anastomosis (IPAA), which can be performed as a two-stage procedure (proctocolectomy with IPAA and diverting ileostomy followed by ileostomy closure) or as a three-stage procedure (subtotal colectomy with subsequent ileostomy, then completion proctectomy, IPAA and diverting ileostomy and as last stage ileostomy closure). The three-stage approach is preferred in high-risk patients with severe inflammation of the colon or those undergoing high-dose steroid or biological therapy and nowadays the more common procedure^5,6^. However, the choice for a 2-stage versus 3-stage procedure is discussed controversially.

Previous studies demonstrated that surgical management of ulcerative colitis offers good functional results and a high patient satisfaction, even if it is associated with a relevant morbidity (30–70%)^7–10^. However, data about the outcome after IPAA stratified to 2- versus 3-stage procedures are limited and showed heterogeneous results, especially regarding long-term outcomes. A severe long-term complication after IPAA is pouch failure, which is reported to occur in 1.6% to 18.9% of patients^11^. The most frequently documented risk factors for pouch failure are major postoperative complications including perioperative pelvic sepsis and anastomotic leakage indicating the importance of IPAA performance in high-volume centers^11–14^. However, the impact of the specific procedure type (2-stage versus 3-stage) on pouch failure during long-term follow-up has not been sufficiently elucidated.

Therefore, the aim of this study was to provide current data on short- and long-term complications after 2- versus 3-stage IPAA in patients with UC and to identify pre-, peri- and postoperative risk factors for morbidity, longer hospital stay and readmission during short-term follow-up as well as for pouch failure and failure of improved quality of life during long-term follow-up.

## Results

### Demographics

176 patients (median age 54 years, 39% female) met the inclusion criteria. 131 of them (74%) received a 2-stage procedure and 45 (26%) a 3-stage procedure (Table 1). Patients who underwent a 3-stage procedure had a significantly lower age (40 vs. 56 years, *p* > 0.001) and lower BMI (22.2 vs. 23.6 kg/m^2^, *p* = 0.008), a significantly shorter duration of disease (52 vs. 96 months, *p* = 0.002) and a shorter conservative treatment time (30 vs. 64 months, *p* = 0.004), had significantly less frequent hypertension (7 vs. 24%, *p* = 0.015) as well as were significantly more frequently female (56 vs. 33%, *p* = 0.008) (Table 1).

**Table 1:** Patient demographics and disease characteristics of patients undergoing IPAA and stratified to the surgical approach (Two-stage vs. Three-stage IPAA).

	All patients	Two-stage IPAA	Three-stage IPAA	*p*-value
Number	176	131 (74)	45 (26)	
Age (years), median (IQR)	54 (24)	56 (19)	40 (24)	** < 0.001**
Gender, n (%)				**0.008**
Female	68 (39)	43 (33)	25 (56)	
Male	108 (61)	88 (67)	20 (44)	
ASA, n (%)				0.475
I/II	144 (84)	108 (86)	36 (80)	
III/IV	27 (16)	18 (14)	9 (20)	
BMI (kg/m^2^), median (IQR)	23.6 (6.1)	24.1 (6.1)	22.2 (5.8)	**0.036**
Comorbidity, n (%)				
Hypertension	34 (19)	31 (24)	3 (7)	**0.015**
PSC	30 (17)	23 (18)	7 (16)	0.823
Diabetes	14 (8)	11 (8)	3 (7)	0.767
Cardiovascular	6 (3)	6 (5)	0 (0)	0.203
Previous abdominal surgery, n (%)	69 (39)	55 (42)	14 (31)	0.219
Previous perineal surgery, n (%)	17 (10)	16 (12)	1 (2)	0.075
Duration of disease (months), median (IQR)	89 (130)	96 (135)	52 (115)	**0.002**
Duration of conservative treatment (months), median (IQR)	52 (89)	64 (95)	30 (40)	**0.004**
Preoperative symptoms				
Bowel movements per day, median (IQR)	12 (12)	12 (12)	15 (12)	0.668
Abdominal pain, n (%)	32 (18)	20 (15)	12 (27)	0.117
Hemtaochezia, n (%)	75 (43)	52 (40)	23 (51)	0.222

IPAA, Ileal Pouch-Anal Anastomosis; ASA, American Society of Anesthesiologists classification; BMI, body mass index; PSC, primary sclerosing cholangitis.

Significant values are in bold.

### Surgical parameters

The primary indication for surgery in our cohort was the failure of conservative medical management (71%), followed by cases involving dysplasia or malignancy (23%). Emergency surgery was performed in 6% of the patients. Patients undergoing a 3-stage procedure had a significantly higher frequency of urgent surgery (20 vs. 1%, *p* < 0.001), with toxic megacolon or perforation being the primary indications. Furthermore, within the three-stage procedure, laparoscopic approach was more frequently performed, especially for the first stage of colectomy (62 vs. 7%, *p* < 0.001) (Table 2).

**Table 2:** Surgical details of patients undergoing IPAA and stratified to the surgical approach (Two-stage vs. Three-stage IPAA).

	All patients (n = 176)	Two-stage IPAA (n = 131)	Three-stage IPAA (n = 45)	*p*-value
Indiction for surgery, n (%)				** < 0.001**
Failure of medical management	125 (71)	91 (70)	34 (76)	
Carcinoma	26 (15)	22 (17)	4 (9)	
Dysplasia	14 (8)	14 (11)	0 (0)	
Toxic megacolon	5 (3)	0 (0)	5 (11)	
Perforation	4 (2)	2 (2)	2 (4)	
Stricture	2 (1)	2 (2)	0 (0)	
Urgent procedure, n (%)	10 (6)	1 (1)	9 (20)	** < 0.001**
Any stage in laparoscopic technique, n (%)	37 (21)	9 (7)	28 (62)	** < 0.001**
Severe inflammation in specimen, n (%)	154 (88)	113 (86)	41 (91)	0.449

IPAA, Ileal Pouch-Anal Anastomosis.

Significant values are in bold.

### Outcome parameters

Regarding short-term outcome, in-hospital morbidity and rate of unplanned return to theatre after any stage were 69% and 10%, respectively. Median length of hospital stay for all stages together was 17 days, with a significantly longer length in the 3-stage-procedure-group (22 vs. 16 days, *p* < 0.001) (Table 3). During 90-day follow-up, 26% required readmission, the anastomotic leakage rate was 11%.

**Table 3:** Outcome parameter of patients undergoing IPAA and stratified to the surgical approach (Two-stage vs. Three-stage IPAA).

		All patients (n = 176)	Two-stage IPAA (n = 131)	Three-stage IPAA (n = 45)	*p*-value
Short-term outcome (n = 176)					
In-hospital	Any morbidity after any stage, n (%)	122 (69)	87 (66)	35 (78)	0.298
	Anastomotic leakage, n (%)	11 (6)	10 (8)	1 (2)	0.294
	Stomata complications, n (%)	15 (9)	8 (6)	7 (16)	0.064
	SSI, n (%)	6 (3)	5 (4)	1 (2)	0.694
	Paralysis/ileus, n (%)	18 (10)	12 (9)	6 (13)	0.569
	Any re-operation after any stage, n (%)	18 (10)	15 (12)	3 (7)	0.414
	Total length of hospital stay for all stages together (days), median (IQR)	17 (8)	16 (8)	22 (12)	** < 0.001**
90-days-follow-up	Any readmission within 90 days after any stage, n (%)	46 (26)	30 (23)	16 (36)	0.116
	Anastomotic leakage, n (%)	19 (11)	15 (11)	4 (9)	0.784
Long term outcome (n = 137)					
Long-term follow-up	Follow-up time (months), median (IQR)	102 (116)	130 (98)	37 (52)	** < 0.001**
	Pouch failure, n (%)	13 (10)	12 (12)	1 (3)	0.112
	Pouchitis, n (%)	63 (46)	46 (46)	17 (45)	1.000
	Anal stricture, n (%)	17 (12)	12 (12)	5 (13)	1.000
	Perianal fistula or abscess, n (%)	24 (18)	21 (21)	3 (8)	0.081
	Bowel movements per day, median (IQR)	8 (6)	8 (7)	8 (5)	0.729
	Ileus, n (%)	21 (15)	17 (17)	4 (11)	0.432
	Incision hernia, n (%)	29 (21)	20 (20)	9 (24)	0.816
	Quality of life impoved by surgery? (n = 117), n (%)				0.672
	Yes	105 (90)	72 (89)	33 (92)	
	Undicided	3 (3)	3 (4)	0 (0)	
	No	9 (8)	6 (7)	3 (8)	

IPAA, Ileal Pouch-Anal Anastomosis; SSI, Surgical Site Infection.

Significant values are in bold.

Median long-term follow-up was 102 months. Pouch failure occurred in 13 patients (10%). During follow-up 46%, 12% and 18% of the patients suffered from pouchitis, anal stricture and perianal fistula or abscess. The median bowel movement was significantly reduced compared to the preoperative status and was in median 8 times per day. Ileus and incisional hernia during follow-up were present in 15% and 21%, respectively. 90% of the patients who were contacted by telephone reported that their quality of life was significantly improved by surgical management, whereas 8% of patients had no improvement and 3% were undecided (Table 3). Except for the duration of hospital stay, all short- and long-term outcome parameters did not differ between the 2-stage and the 3-stage groups (Table 3).

### Short-term outcome

No significant risk factor could be identified in the univariate analysis for the occurrence of any in-hospital morbidity after any stage, thus precluding the need for multivariate analysis (Table 4).

**Table 4:** Risk factors for postoperative in-hospital morbidity after any stage, longer length of hospital stay (all stages together) and 90-days readmission after any stage in patients undergoing IPAA (n = 176).

		Any in-hospital morbidity				Longer length of hospital stay (> 17 days)				Any readmission (within 90 days postoperatively)			
		Univariate	Multivariate			Univariate	Multivariate			Univariate	Multivariate		
			HR	95% CI	*p*		HR	95% CI	*p*		HR	95% CI	*p*
Preoperative parameters	Age (≤ 50 vs. > 50 years)*	0.584	–	–	–	0.449	–	–	–	0.863	–	–	–
	Gender (female vs. male)	1.000	–	–	–	0.217	–	–	–	0.381	–	–	–
	ASA (I/II vs. III/IV)	0.453	–	–	–	0.062	–	–	–	0.098	–	–	–
	BMI (≤ 25 vs. > 25 kg/m^2^)*	0.852	–	–	–	0.087	–	–	–	0.859	–	–	–
	Hypertension (yes vs. no)	0.507	–	–	–	0.850	–	–	–	0.388	–	–	–
	PSC (yes vs. no)	1.000	–	–	–	0.422	–	–	–	0.497	–	–	–
	Diabetes (yes vs. no)	1.000	–	–	–	0.168	–	–	–	0.200	–	–	–
	Cardiovascular (yes vs. no)	0.615	–	–	–	0.222	–	–	–	0.690	–	–	–
	Previous abdominal surgery (yes vs. no)	1.000	–	–	–	0.878	–	–	–	0.598	–	–	–
	Previous perianal surgery (yes vs. no)	0.064	–	–	–	1.000	–	–	–	0.565	–	–	–
	Duration of disease (≤ 89 vs. > 89 months)	0.357	–	–	–	0.355	–	–	–	0.164	–	–	–
	Duration of conservative treatment (≤ 52 vs. > 52 months)*	0.812	–	–	–	1.000	–	–	–	1.000	–	–	–
Surgical parameters	Indication for surgery (inflammation vs. dysplasia/malignancy)	0.196	–	–	–	0.282	–	–	–	** < 0.001**	**9.5**	**1.9**–**48.5**	**0.007**
	Urgent procedure (yes vs. no)	0.694	–	–	–	**0.006**	7.6	0.7–78.3	0.090	**0.021**	**7.8**	**1.7**–**36.1**	**0.009**
	Laparoscopic parts of surgery (yes vs. no)	0.075	–	–	–	**0.026**	1.0	0.3–2.8	0.971	1.000	–	–	–
	Severe inflammation in specimen (yes vs. no)	0.274	–	–	–	1.000	–	–	–	0.199	–	–	–
	Kind of IPAA procedure (2-stage vs. 3-stage)	0.298	–	–	–	** < 0.001**	**3.8**	**1.3**–**10.8**	**0.014**	0.116	0.8	0.3–2.2	0.704
Short-term follow-up parameters	Any morbidity (yes vs. no)					**0.006**	**3.3**	**1.4**–**7.9**	**0.008**	** < 0.001**	**7.5**	**1.5**–**37.8**	**0.015**
	In-hospital anastomotic leakage (yes vs. no)					0.349	–	–	–	**0.037**	2.8	0.8–10.5	0.117
	Stomata complications (yes vs. no)					1.000	–	–	–	0.762	–	–	–
	SSI (yes vs. no)					**0.008**	**	**	**	1.000	–	–	–
	Paralysis/ileus (yes vs. no)					0.456	–	–	–	** < 0.001**	**9.3**	**2.7**–**32.2**	** < 0.001**
	Any resurgery (yes vs. no)					0.456	–	–	–	0.087	–	–	–
	Length of hospital stay > 17 days (yes vs. no)									0.087	–	–	–

ASA, American Society of Anesthesiologists classification; BMI, Body Mass Index; PSC, primary sclerosing cholangitis; IPAA, Ileal Pouch-Anal Anastomosis; SSI, Surgical Site Infection.

*Cut-off = median.

**Parameter constant > no inclusion to multivariate analysis.

Significant values are in bold.

In univariate analysis, several factors were significantly associated with a longer length of hospital stay, including the need for an urgent procedure (90% vs. 43%, *p* = 0.006), the presence of laparoscopic parts of surgery (62% vs. 41%, *p* = 0.026), the 3-stage procedure (73% vs. 36%, *p* < 0.001), the occurrence of any morbidity (51% vs. 26%, *p* = 0.006), and surgical site infection (100% vs. 44%, *p* = 0.008). In the multivariate analysis, the 3-stage procedure (OR 3.8, 95% CI 1.3–10.8, *p* = 0.014) and the occurrence of any morbidity (OR 3.3, 95% CI 1.4–7.9, *p* = 0.008) were confirmed as independent risk factors for a longer hospital stay (Table 4).

The presence of inflammation as indication for surgery (32% vs. 5%, *p* < 0.001), the need for an urgent procedure (60% vs. 24%, *p* = 0.021), the occurrence of any morbidity (32% vs. 5%, *p* < 0.001), anastomotic leakage (55% vs. 24%, *p* = 0.037), and postoperative paralysis/ileus (67% vs. 22%, *p* < 0.001) were significantly associated with the need for readmission within 90 days postoperatively. Multivariate analysis revealed that the presence of inflammation as an indication for surgery (OR 9.5, 95% CI 1.9–48.5, *p* = 0.007), the need for an urgent procedure (OR 7.8, 95% CI 1.7–36.1, *p* = 0.009), the occurrence of any morbidity (OR 7.5, 95% CI 1.5–37.8, *p* = 0.015) and postoperative paralysis/ileus (OR 9.3, 95% CI 2.7–32.2, *p* < 0.001) were independent risk factors for the need of readmission within 90 days postoperatively (Table 4). The kind of procedure (2-stage versus 3-stage approach) did not reach statistical significance in either univariate or multivariate analyses.

### Long-term outcome

In univariate analysis, several factors showed a higher rate of pouch failure, including hypertension (23% vs. 6%, *p* = 0.017), primary sclerosing cholangitis (25% vs. 7%, *p* = 0.023), previous abdominal surgery (22% vs. 2%, *p* < 0.001), need for re-surgery (31% vs. 7%, *p* = 0.008), longer hospital stay (15% vs. 4%, *p* = 0.045), 90-days anastomotic leakage (50% vs. 4%, *p* < 0.001), occurrence of pouchitis during follow-up (16% vs. 4%, *p* = 0.021) and presence of perianal fistula or abscess during follow-up (38% vs. 4%, *p* < 0.001). Among these factors, only the occurrence of perianal fistula or abscess during follow-up (OR 13.0, 95% CI 1.9–91.3, *p* = 0.010) was confirmed as independent risk factor for pouch failure in the multivariate analysis (Table 5). Pouch failure was not influenced by the kind of procedure (2-stage versus 3-stage approach) in uni- and multivariate analysis.

**Table 5:** Risk factors for long-term pouch failure during follow-up (n = 137) and fail of improved quality of life during follow-up (n = 117) in patients undergoing IPAA.

		Pouch failure				Fail of improved quality of life			
		Univariate	Multivariate			Univariate	Multivariate		
			HR	95% CI	*p*		HR	95% CI	*p*
Preoperative parameters	Age (≤ 50 vs. > 50 years)*	1.000	–	–	–	0.550	–	–	–
	Gender (female vs. male)	0.237	–	–	–	0.761	–	–	–
	ASA (I/II vs. III/IV)	0.220	–	–	–	0.087	–	–	–
	BMI (≤ 25 vs. > 25 kg/m^2^)*	0.080	–	–	–	0.761	–	–	–
	Hypertension (yes vs. no)	**0.017**	5.3	0.8–35.1	0.085	0.216	–	–	–
	PSC (yes vs. no)	**0.023**	1.0	0.1–7.9	0.966	0.708	–	–	–
	Diabetes (yes vs. no)	0.599	–	–	–	0.596	–	–	–
	Cardiovascular (yes vs. no)	0.259	–	–	–	1.000	–	–	–
	Previous abdominal surgery (yes vs. no)	** < 0.001**	9.8	1.0–97.1	0.051	1.000	–	–	–
	Previous perianal surgery (yes vs. no)	0.089	–	–	–	0.599	–	–	–
	Duration of disease (≤ 89 vs. > 89 months)	0.535	–	–	–	0.765	–	–	–
	Duration of conservative treatment (≤ 52 vs. > 52 months)*	0.193	–	–	–	0.187	–	–	–
Surgical parameters	Indication for surgery (dysplasia vs. inflammation)	0.297	–	–	–	0.066	–	–	–
	Urgent procedure (yes vs. no)	0.608	–	–	–	0.599	–	–	–
	Laparoscopic parts of surgery (yes vs. no)	0.733	–	–	–	0.470	–	–	–
	Severe inflammation in specimen (yes vs. no)	0.694	–	–	–	0.698	–	–	–
	Kind of IPAA procedure (2-stage vs. 3-stage)	0.112	0.1	0.0–2.8	0.182	0.752	1.1	0.2–5.0	0.927
Short-term follow-up parameters	Any morbidity (yes vs. no)	0.462	–	–	–	0.458	–	–	–
	Stomata complications (yes vs. no)	1.000	–	–	–	1.000	–	–	–
	SSI (yes vs. no)	0.071	–	–	–	0.083	–	–	–
	Paralysis/ileus (yes vs. no)	0.360	–	–	–	0.159	–	–	–
	Any resurgery (yes vs. no)	**0.008**	1.9	0.2–24.0	0.617	0.109	–	–	–
	Length of hospital stay > 17 days (yes vs. no)	**0.045**	3.0	0.4–22.0	0.288	0.131	–	–	–
	Any readmission (yes vs. no)	0.188	–	–	–	0.097	–	–	–
	90-days anastomotic leakage (yes vs. no)	** < 0.001**	5.9	0.8–45.5	0.086	1.000	–	–	–
Long-term follow-up parameters	Pouchitis (yes vs. no)	**0.021**	4.4	0.6–32.5	0.147	0.544	–	–	–
	Anal stricture (yes vs. no)	0.369	–	–	–	0.636	–	–	–
	Perianal fistula or abscess (yes vs. no)	** < 0.001**	**13.0**	**1.9**–**91.3**	**0.010**	1.000	–	–	–
	Ileus (yes vs. no)	0.113	–	–	–	**0.015**	4.2	1.0–17.4	0.051
	Incision hernia (yes vs. no)	0.472	–	–	–	1.000	–	–	–
	Pouch failure (yes vs. no)					**0.002**	**13.0**	**2.3**–**74.7**	**0.004**

ASA, American Society of Anesthesiologists classification; BMI, Body Mass Index; PSC, primary sclerosing cholangitis; IPAA, Ileal Pouch-Anal Anastomosis; SSI, Surgical Site Infection.

*Cut-off = median.

Significant values are in bold.

The occurrence of ileus (29% vs. 7%, *p* = 0.015) and pouch failure (57% vs. 7%, *p* = 0.002) during follow-up were associated with a failure to improve quality of life in the univariate analysis. In the multivariate analysis, pouch failure remained a significant independent risk factor for the failure to achieve improved quality of life (OR 13.0, 95% CI 2.3–74.7, *p* = 0.004) (Table 5). The kind of procedure (2-stage versus 3-stage approach) failed to be significant in uni- and multivariate analysis.

## Discussion

Restorative proctocolectomy with ileal pouch-anal anastomosis (IPAA) has been established as the standard treatment for patients with therapy-refractory, malignant or complicated ulcerative colitis (UC). However, data regarding the optimal procedure type (2-stage versus 3-stage approach) are limited and discussed controversially.

In our single-center analysis of 176 consecutive patients who underwent either 2- or 3-stage restorative proctocolectomy with ileal pouch-anal anastomosis (IPAA) for ulcerative colitis, favorable long-term outcomes were observed. The majority of patients reported significant improvements in their quality of life after the operation. Surgical procedures were associated with a relevant, but acceptable morbidity, which can be attributed to the multiple stages and complexity of the surgical intervention.

The 3-stage procedure was associated with a longer hospital stay, which can be explained by the additional third hospitalization. Additionally, there were notable differences in the patient characteristics between the two groups. Patients who underwent the 3-stage procedure were generally younger, had a lower body mass index (BMI), shorter medical history of ulcerative colitis and had a higher frequency of emergency surgery due to toxic megacolon or perforation. These factors align with our indication for utilizing the 3-stage approach specifically in cases of severe ulcerative colitis. However, apart from the influence on hospital stay and patient characteristics, the 3-stage procedure did not demonstrate any significant impact on other outcome parameters. These findings are consistent with previous studies that have investigated effects of the 2- versus 3-stage procedure on various outcome parameters^10,16^.

In terms of short-term outcome, morbidity rates in our cohort, including anastomotic leakage, re-surgery, and readmission, were comparable to those reported in other studies when considering surgeries of any stage collectively, as we did in our study^10,17^. It is important to note that many comparable studies only focus on isolated short-term outcome parameters of the IPAA procedure, which may initially suggest a lower complication rate. The median length of hospital stay for all stages was slightly higher compared to other studies, which may be attributed to differences in healthcare systems^10^.

In our cohort, we did not identify any specific risk factors for the occurrence of in-hospital morbidity after any stage, which is consistent with findings from other authors^18^. However, previous studies have reported factors such as advanced age, higher body mass index (BMI) and open surgery as associated with higher morbidity rates, none of which could be confirmed in our cohort^10,19^.

To our knowledge, the outcome parameter of a longer hospital stay has not been extensively investigated before. In our cohort, we identified two independent risk factors for a longer hospital stay: the 3-stage procedure, as previously mentioned, and the occurrence of any complication, which necessitates extended and more intensive care, resulting in a prolonged hospital stay.

Regarding the need for readmission within the first 90 days postoperatively, the presence of inflammation as an indication for surgery, the need for an urgent procedure, the occurrence of any morbidity and a postoperative ileus were identified as independent risk factors. While these parameters were not explicitly described in the literature, they appear to be reasonable. McKenna et al. identified a BMI > 30 kg/m^2^ as a protective factor against readmission^20,21^; however, BMI did not have a significant impact on the readmission rate in our analysis.

The results of our long-term analysis, with a pouch failure rate of 10% and a 90% improvement in quality of life, are consistent with data in the existing literature. A recent meta-analysis has reported pouch failure rates ranging from 1.6 to 18.9%, our findings are located in the middle of this range^11^. However, when comparing pouch failure rates among different studies, it is crucial to consider the follow-up duration, as some studies have demonstrated a continuous increase in the rate even after several years^12,14^. Mark-Christensen et al. reported a cumulative risk of pouch failure rate at 10 years of follow-up of 12.1%, which is similar to our findings (10% after 8.5 years)^14^.

Numerous risk factors for pouch failure have been documented in the literature, with pelvic sepsis—mostly due to anastomotic leakage—being the most commonly reported factor^12,22,23^. Additionally, chronic pouchitis, fistulas, postoperative diagnosis of Crohn's disease, preoperative primary sclerosing cholangitis, female gender, perioperative use of biological agents and necessary pouch revision are reported factors associated with pouch failure^11–14^. Our analysis confirms the occurrence of perianal fistula or abscess as an independent risk factor for pouch failure. PSC, anastomotic leakage and pouchitis during follow-up were also associated with a higher rate of pouch failure in univariate analysis in our cohort, without significance in multivariate analysis. Perianal fistulas and abscesses can be attributed to Crohn's disease in some cases. However, a thorough review of histological findings, including pouch explant specimens, did not indicate any presence of Crohn's disease in our patients with pouch failure. It should be noted that the causes of perianal fistulas and abscesses can be diverse, including factors as anastomotic leaakage, pouchitis or other inflammatory processes. Therefore, our data, in conjunction with the existing literature, could be interpreted that the presence of an inflammatory process surrounding the pouch, irrespective of its origin (anastomotic insufficiency, abscess, fistula, or pouchitis—all factors, which were significantly associated with pouch failure in univariate analysis), appears to be the primary risk factor for pouch failure.

Notably, in our analysis, pouch failure emerged as the most significant and the only independent risk factor for the failure to achieve improved quality of life after surgical management, emphasizing the importance of this long-term outcome parameter.

One of the strengths of our analysis is the considerable median follow-up time of over 8 years after the performance of IPAA. However, it is important to acknowledge the limitations of our study. Firstly, the retrospective design introduces the possibility of inherent biases and limitations associated with such an approach. Secondly, the patient cohort was recruited over a lengthy time period, during which there may have been changes in medical treatment options and practices, potentially impacting the characteristics of the patient population. Lastly, we did not employ a validated and structured quality of life assessment tool, which would have significantly enhanced the robustness and reliability of our quality of life data.

## Conclusion

Our analysis confirms that restorative proctocolectomy with IPAA is an effective treatment option with acceptable morbidity for ulcerative colitis, yielding favorable long-term results and high patient satisfaction. Although the 3-stage procedure may lead to a longer hospital stay, it does not appear to have a substantial impact on other outcomes when compared to the 2-stage procedure.

## Materials and methods

We retrospectively analyzed 176 consecutive patients with ulcerative colitis (UC) who received proctocolectomy with an ileal pouch-anal anastomosis (IPAA) as either 2-stage or 3-stage procedure from 2001 to 2021 at the University Hospital Erlangen. Patients had to meet following inclusion criteria: (1) age of 18 years or older; (2) histologically proven ulcerative colitis (UC); (3) performance of IPAA as a 2-stage or 3-stage procedure. Both open and laparoscopic approaches and both elective and emergency surgeries were included.

Data on patient demographics, comorbidities, preoperative parameters and surgical technique as well as on the postoperative course were obtained from our clinical data system. Additionally, attempts were made to contact each patient by telephone in order to obtain updated follow-up information. This enabled the collection of current follow-up data from 137 patients (78%). Data were analyzed for all patients and stratified by the 2-stage and the 3-stage approach.

Primary endpoint was the incidence of pouch failure during long-term follow-up. Secondary endpoints were the incidence of in-hospital morbidity, longer hospital stay and 90-days-readmission as well as failure of improved quality of life during long-term follow-up.

This study was approved by the Ethics Committee of FAU Erlangen (22-222-Br). The work was registrated at the German Clinical Trial Registry (DRKS00033015) and reported in line with the STROBE criteria. This study, including all methods, was performed in accordance with the current Declaration of Helsinki and with the standards of Good Clinical Practice (GCP).

### Surgical techniques

All patients received either a 2-stage or 3-stage procedure. The 2-stage procedure included, as the first step, a proctocolectomy with IPAA creation and diverting ileostomy and as the second step, a closure of the diverting ileostomy. The 3-stage procedure included, as the first step, a subtotal colectomy with an end ileostomy; as the second step, a completion proctectomy with IPAA construction and diverting ileostomy; and as the third step, the closure of the diverting ileostomy. All pouches were performed in a J-pouch configuration. All ileo-anal anastomoses were stapled, no hand-sewn pouch-anal anastomoses were performed. The diverting ileostomy is typically closed three months after J-pouch reconstruction.

### Definitions

Morbidity was defined as any deviation from the normal postoperative course according to the Clavien-Dindo classification^15^ and was assessed during hospital stay (in-hospital morbidity). Any morbidity in the postoperative course of any stage was considered for the outcome parameter “In-hospital morbidity”. Patients with a hospital stay duration greater than the median duration of all stages combined (17 days) were classified as having a “longer hospital stay”. 90-days readmission was defined as any readmission occurring within the first 90 postoperative days after any stage. Anastomotic leakage was assessed during in-hospital stay and within 90 days postoperatively. Pouch failure was defined as pouch excision and creation of a permanent stoma or the need of a permanent diversion stoma. All patients who indicated during telephone follow-up that they did not experience any improvement in quality of life from the surgical procedure or were undecided were categorized as having a failed improvement of quality of life.

### Statistical analysis

Data analysis was performed with SPSS software (SPSS, version 28.0). Comparisons of metric and ordinal data were calculated with the Student’s t-test or Mann Whitney U test. The Chi-square test was used for categorical data. Statistical significance was set at *p* < 0.05. All recorded parameters were tested as potential risk factors for short-term outcome parameters (morbidity, longer hospital stay, re-admission) and long-term outcome parameters (pouch failure, failure of improved quality of life) using univariate analysis. Associations with the outcome parameters with a *p*-value ≤ 0.05 in univariate analysis as well as the kind of procedure (2-stage versus 3-stage approach) were included in multivariate analysis.

## Data Availability

All data underlying this article are available in the article.

## Statement on the welfare of animals

This article does not contain any studies with animals performed by any of the authors.
